# Comparison of the fungicidal efficacy of photodynamic therapy with methylene blue, silver nanoparticle, and their conjugation on oral *Candida* isolates using cell viability assay

**DOI:** 10.18502/cmm.6.4.5332

**Published:** 2020-12

**Authors:** Fatemeh Lavaee, Motahare Yousefi, Pardis Haddadi

**Affiliations:** 1 Oral and Dental Disease Research Center, Oral and Maxillofacial Medicine Department, Faculty of Dentistry, Shiraz University of Medical Sciences, Shiraz, Iran; 2 Alborzi Clinical Microbiology Research Center, Shiraz University of Medical Sciences, Shiraz, Iran; 3 Student Research Committee, School of Dentistry, Shiraz University of Medical Sciences, Shiraz, Iran; 4 Department of Periodontology, Faculty of Dentistry, Lorestan University of Medical Sciences, Khorramabad, Iran

**Keywords:** *Candida albicans*, *Candida parapsilosis*, *Candida glabrata*, Photodynamic therapy, Silver nanoparticle

## Abstract

**Background and Purpose:**

This study aimed to evaluate the effect of common photodynamic therapy and photodynamic therapy by the silver nanoparticle, methylene blue, and their combination on biofilm and plankton cells of standard oral *Candida* isolates using cell viability assay.

**Materials and Methods:**

In this *in vitro* study, biofilm and plankton cells of *Candida* species(i.e. *C .albicans* and *C. parapsilosis*) and plankton cells of *Candida glabrata* were treated with methylene blue, silver nanoparticle, and their combination once alone and then with the irradiation of total dose of 1.92 J/cm² for 60 sec. The minimum inhibitory concentration and antifungal activity of each approach were evaluated using the XTT assay.

**Results:**

After photodynamic therapy, methylene blue showed antifungal effect only on *Candida albicans*, while the antifungal effect of silver nanoparticles was increased on all *Candida* species. On the other hand, photodynamic therapy with the combination of methylene blue and silver nanoparticles did not have any effect on *C. albicans*. However, it reduced the minimum inhibitory concentration value of *C. parapsilosis*, and the most antifungal effect was observed on *C. glabrata*.

**Conclusion:**

Photodynamic therapy with photosensitizers can serve as a treatment modality in *Candida* infections of the oral cavity. Antifungal effect of photodynamic therapy was strain- and photosensitizer-dependent.

## Introduction

*Candida* species are among the normal flora of the oral cavity and may become pathogenic, especially in patients with immunodeficiency [ 1 - 3 ]. *Candida albicans* is responsible for most oropharyngeal and fungal infections [ 4 - 6 ]. According to the reviewed literature, the prescribed systemic and local antifungal agents can cause the resistance of *Candida* species [ 7 , 8 ]. There are several factors related to resistance to antifungal medications, such as intrinsic resistance of the organism, exposure to antifungal agents for a long period, and the ability of *Candida* to form biofilm on the surfaces [ 9 , 10 ]. 

*Candida* species have produced biofilms on different surfaces, including mucosal cells, dentures, catheters, with *C. albicans* having the greatest potential to form biofilms [ 11 ]. Due to the density of the cells and the presence of the matrix, biofilms have more resistance against antifungal agents in comparison to planktonic cells [ 12 ]. 

Given the insensitivity of micro-organisms to conventional medications and their possible side effects, it is suggested to use new antimicrobial approaches. In photodynamic therapy, light with special wavelengths can produce hydroxyl free radical and singlet oxygen which can lyse the membrane and damage the cells [ 13 , 14 ]. Methylene blue is a potential photosensitizer thiazine dye and can transfer the absorbed energy to molecules to activate photodynamic therapy [ 15 , 16 ]. 

Given the antimicrobial properties of nanoparticles, silver nanoparticles have recently received special attention as an antimicrobial and antifungal agent [ 17 , 18 ]. Silver nanoparticles interact with the main parts of cells (membrane or cell wall, DNA, and microbial proteins) and produce intracellular reactive oxygen species. Accumulation of this intracellular production can regulate apoptosis [ 19 ]. Proper antifungal effect against *C. albicans* by gold nanoparticle-mediated photodynamic therapy was reported in the literature [ 20 , 21 ]. To the best of our knowledge, only few studies have addressed the antifungal effects of photodynamic therapy on silver nanoparticles. 

In an *in vitro* study, gold nanoparticles enhanced the photodynamic therapy results with methylene blue for *C. albicans* biofilm inhibition [ 20 ]. In another similar assessment, gold nanoparticle-photosensitizer conjugate-based photodynamic therapy effectively killed both planktonic *C. albicans* cells and its hyphal form in biofilm [ 21 ]. 

Due to the antifungal prescription limitations and presence of non-pharmacological approaches, this study aimed to compare the effect of photodynamic therapy with methylene blue, silver nanoparticles, and their combination on standard oral *Candida* species using the Cell Proliferation Kit II (XTT)(2,3-bis-(2-methoxy-4-nitro-5-sulfophenyl)-2H-tetrazolium-5-carboxanilide) to introduce a new antifungal treatment with limited side effects. 

## Materials and Methods

**Study sample**

The present *in vitro* research was approved by the Ethics Committee of the Shiraz University of Medical Sciences (ethics code: IR.SUMS.REC.1396.S272).This study was conducted on standard species of *C. albicans* PTCC 50027(ATCC 10231), *Candida parapsilosis* (ATCC 22019), and *Candida glabrata* (ATCC 2001). 

**Biofilm preparation**

*Candida* species were cultured on sabouraud dextrose agar (manufactured by Merck, Germany) at room temperature for24-48 h. To increase the fungal cells, they were suspended in 20 ml yeast peptone dextrose and incubated overnight in an orbital shaker (60 rpm) at 30 °C. The tubes were centrifuged at 3000 g for 5 min at 4°C.The harvested cells were washed with 20 ml ice-cold sterile phosphate buffer saline (PBS) (manufactured by Gibco, USA) in triplicate.

Afterward, the sediment was suspended in 10 ml pre-warmed (37 °C) RPMI 1640 medium (manufactured by Sigma, UK)without sodium bicarbonate and with L-glutamine which buffered to pH 7.0 with 0.165 M morpholinepropane sulfonic acid (manufactured by Sigma, Germany).The suspension was diluted 1:1000 and adjusted to the 106 cells/mL in RPMI 1640) with a hematocytometer Neubauer chamber. Biofilms were produced on commercially 96 microtiterflat-bottom cell and tissue culture Plates (Nantong Renon Laboratory Equipment Co., Ltd, Jiangsu, China). Subsequently, 100 μL of a standard *Candida* suspension was transferred into each well, and the plates were incubated for two h at 37°C in a shaker at 75 rpm to allow them to adhere to the surface of the wells. After the adhesion phase, the supernatant was aspirated and each well was washed twice with 150 μL of PBS to remove the loosely adherent cells. A total of 100 μL of RPMI 1640 was added to each washed wells, and the plates were incubated at 37°C in a shaker at 75 rpm. The biofilms were allowed to develop for 24 h and used in different assays. It should be mentioned that all assays were repeated three times. The *C. glabrata* cannot produce biofilms; accordingly, the experiments were performed on the plankton cells. 

**Minimum inhibitory concentration values of *Candida* species**

Minimum inhibitory concentrations (MIC) of methylene blue, silver nanoparticle, and combination were assessed based on the CLSI M27-A3 standard procedure [ 22 ]. A dilution equal to 0.5 McFarland was prepared in normal saline for each *Candida* species and diluted to 1:1000 with RPMI.Afterward, 100 µL of each photosensitizer dilution and 100 µL of the *Candida* species suspensions were added to each well. Serial dilutions were prepared ranging from 100 to 0.195 µl/ml from methylene blue, silver nanoparticles, and combination. In each series, the 11th and 12th wells were considered as positive and negative wells without photosensitizer and yeast suspension, respectively. The plates were incubated at 37°C and their MIC was recorded after 24 h. Furthermore, similar plates were processed and treated with laser. The MIC is defined as the lowest concentration of photosensitizer with a 50% decrease in growth, compared with the positive control group. The mean values recorded in three different experiments are reported as MIC values. 

**Photosensitizer**

The combination included methylene blue as photosensitizer and silver nanoparticles. To obtain a uniform suspension of silver nanoparticles, 200 mg of silver nanoparticles powder (manufactured by Sigma Aldrich, the USA)[ 18 , 23 , 24 ] with particles sized <100 nm and 100 ml normal saline was poured into the tube and sonicated at 200 W for two min in a sonicator device(manufactured by Hielscher, Germany). The concentration of 200 mg/ml of methylene blue was made in phosphate-buffered saline(20 Mm phosphate buffer, 2.7 Mm potassium chloride, 137 Mm sodium chloride; pH 7.4)[ 14 , 16 , 25 ]. 

A combination of silver nanoparticles and methylene blue with a concentration of 200 mg/ml was prepared. In addition, 10-fold serial dilutions were prepared ranging from 100 to 0.195 µl/ml from methylene blue, silver nanoparticles, and their combination. Biofilms were treated with 100 µl of each concentration for 30 min in darkness (under a laminar flow hood) under two conditions, i.e. with and without photodynamic therapy. Those that were not exposed to laser light or photosensitizer were considered as the positive control group (n=10). Finally, the vitality of *Candida* biofilms was assessed using XTT. 

**Light exposure**

In the pilot study, we examined the efficacy of photodynamic therapy on the yeasts in durations of 1-5 min since it was difficult for patients to tolerate the procedure for more than five min. There was no difference between the applications of laser for different durations in terms of inducing a reduction in viable *Candida* species; therefore, one-min irradiation was applied in this study. Moreover, the light source gallium–aluminum–arsenide laser (manufactured by Easy-Laser, Clean Line, Brazil) was employed. The total output power was 0.025 W, and the continuous mode of the device with 660 nm was used. The cross-section of the laser probe was 0.78 cm², and all *Candida* preparations were irradiated with a total dose of 1.92 J/cm² for 60 sec. 

**The Cell Viability Reduction assay**

The XTT, which is a colorimetric assay system, was used to reassess the susceptibilities of the *Candida* species to photosensitizer since the viability of the cells was not clear by spectrophotometric readings. The XTT solution was prepared as a saturated solution at 0.5 mg/ml in sterile ringer lactate and covered with aluminum foil during preparation. After filtering it with a 0.22 µm pore size filter, the XTT solution was aliquoted into 10 ml working volume and stored at -70°C. Afterward, 10 Mm stock solution of menadione in 100% acetone was prepared, liquated into smaller volumes (about 50 µm), and stored at -70°C. Moreover, tubes containing 10 ml of the XTT solution were thawed (one per plate) as required for the experimental design.

Subsequently, 1µl of the stock solution of menadione was added to each tube to achieve a final menadione concentration of 1 µM. Besides, 100 µl XTT/menadione solution was added to each well, and the plate was covered with aluminum foil and incubated under the dark condition at 37°C on a rotating shaker. After one h, the plates were uncovered, and 75-80 µl of the colored supernatant of each well was transferred to a new plate. The plates were measured at 475 and 660 nm with a spectrophotometer, and the wells without biofilms were considered as a blank. 

Absorbance of each sample was evaluated using the following formula according to the XTT assay kit: Specific Absorbance=A475nm (Test) – A475nm (Blank) – A660nm (Test) 

## Results

Sensitivity results of the photosensitizer (i.e., methylene blue, silver nanoparticles, and their combination) were different in various *Candida* species. As shown in Table 1, the reported MICs (plankton cells) for methylene blue were the same before and after irradiation (100 µg/ml) in *C. parapsilosis*, while the silver nanoparticles and combination MICs decreased after illumination. The MIC values of both silver nanoparticles and combination in *C. parapsilosis* were 50 and 25 before and after irradiation, respectively. Viability of biofilm formation (by XTT test) of *C. parapsilosis* in silver nanoparticles and combination decreased after irradiation by 30% and 50%, respectively. Methylene blue showed no anti-*C. parapsilosis* effects in the biofilm and plankton cells before and after irradiation. 

**Table 1:** Minimum inhibitory concentration (MIC) and results of cell viability assay of Candida species before and after irradiation with methylene blue, silver nanoparticle, and their combination

Organism		Methylene Blue	Silver Nano Particle	Combination
*Candida parapsilosis*	MIC (µg/ml)			
	Before	100	50	50
	After	100	25	25
	Cell viability after irradiation	No reduction	30% reduction after laser illumination	50% reduction after laser illumination
*Candida albicans*	MIC (µg/ml)			
	Before	100	50	100
	After	50	25	100
	Cell viability after irradiation	10% reduction after laser illumination	50% reduction after laser illumination	15% reduction after laser illumination
*Candida glabrata*	MIC (µg/ml)			
	Before	100	50	25
	After	100	25	12.5
	Cell viability after irradiation	No reduction	20% reduction after laser illumination	50% reduction after laser illumination

The MICs of Methylene blue and the combination for *C. albicans* (100 µg/ml) and silver nanoparticles (50 µg/ml) were the same before irradiation (Table 1). However, after photodynamic therapy, the MIC values of Methylene blue and silver nanoparticle underwent a decrease. *In vitro* viability evaluation of *C. albicans* by the XTT method revealed 15%, 50%, and 10% viability reduction after photodynamic therapy in the yeasts treated with Methylene blue, silver nanoparticle, and the combination, respectively. Lowest MIC value of *C. albicans*, (25 µg/ml) was observed when yeasts were treated with silver nanoparticle and photodynamic therapy. Methylene blue and the combination that treated *C. albicans* did not show a significant colony-forming unit (CFU) reduction viability in comparison with silver nanoparticles alone. 

Since *C. glabrata* does not have pseudohypha, the anti-biofilm activity could not be evaluated and results for plankton form were reported. The MIC values of Methylene blue for *C. glabrata* before and after photodynamic therapy were the same, while the MICs of silver nanoparticles and combination treatments decreased after photodynamic therapy (50 to 25 and 25 to 12.5). According to the XTT assay, 20% and 50% viability reduction was seen in silver nanoparticle and combination treatment. The lowest (12.5 µg/ml) MIC value for *C. glabrata* was obtained after the treatment of yeasts by the combination and photodynamic therapy.

**Figure 1:**
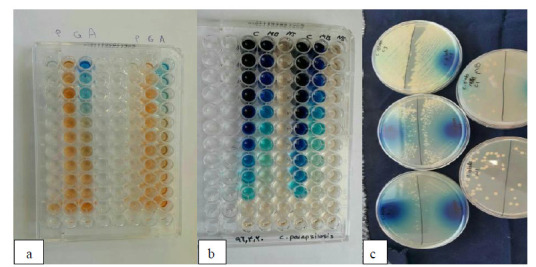
Combination of methylene blue and silver nano-particles

Overall, photodynamic therapy with silver nanoparticles was the most effective treatment for *C. albicans* and *C. parapsilosis* biofilms. Moreover, photodynamic therapy with the combination was the most effective treatment for the inhibition of *C. glabrata* plankton cells. Figure 1 shows the laboratory assessments of XTT assay and MIC determination. 

## Discussion

In the present study, standard species of *C. albicans*, *C. parapsilosis*, and *C. glabrata* were assessed since *Candida albicans* is the major cause of oropharyngeal candidiasis in immunocompromised patients. Furthermore, according to the literature, due to prolonged use of fluconazole in prophylaxis and treatment of the patients, non-albicans *Candida* species like *C. parapsilosis* and *C. glabrata* which are isolated from oral candidiasis are resistant to antifungal agents and non-wild type species [ 26 , 27 ]. In this regard, the use of safe and effective therapy for the treatment of patients is necessary. 

According to the results, photodynamic therapy with methylene blue, silver nanoparticle, and their combination as photosensitizers were effective in reducing the MIC values and viability of some *Candida* species based on the XTT assay. During photodynamic therapy, the cells are damaged by necrosis and apoptosis since the interaction between light and the biological cells produces highly cytotoxic singlet oxygen and other reactive oxygen species [ 28 ]. The role of photosensitizers is to transfer photon energy to the cells and create interaction between a chemical constituent of photosensitizers and a specific wavelength of light [ 29 - 31 ]. 

In this study, methylene blue, silver nanoparticles, and their combination were used as the photosensitizers. Methylene blue promotes cell death by the production of cytotoxic singlet oxygen and other reactive oxygen species [ 14 ].Silver nanoparticle damages the cell membrane by attaching to the sulfur-containing proteins of the cell membrane which decreases the intracellular adenosine 5'-triphosphate level and destroys the microorganism DNA. Besides, the silver nanoparticle has a significant role in mitochondrial dysfunctional apoptosis due to the generated hydroxyl radicals [ 32 ]. Kim et al. found that silver nanoparticle has considerable antifungal activity on *C. albicans* by the pit formation on the cell membrane and ion leakage was confirmed by the transmission electron microscopy image [ 18 ]. 

Usage of photodynamic therapy alone and with different photosensitizers must be synergistic; however, in the present study, different results were observed based on the yeast type. Based on the results of a study performed by Sousaet al., the MIC reduction was significant (*P*˂0.05) for *C. albicans* during five-min incubation with laser with a concentration of 150 μg/mL of methylene blue [ 32 ]. In this study, a one-min photodynamic therapy treatment was selected with the concentration of 100 μg/mL of methylene blue since in higher concentrations the dark color of methylene blue influences the spectrophotometric reading. 

Based on the results of a study carried out by Souza [ 33 ], the MIC values of *C. albicans* for methylene blue decline dafter irradiation and methylene blue was found to be an effective photosensitizer in antifungal photodynamic therapy. These findings are in line with those of the present study. According to the related literature, the antimicrobial properties of methylene blue mediated photodynamic therapy [ 34 , 35 ] and also photosensitizer alone had a non-fungicidal effect on *C. albicans* [ 36 , 37 ]. 

In this study, methylene blue did not have any antifungal effects on *C. glabrata* and *C. parapsilosis* plankton cells before and after the irradiation. Moreover, it was found that silver nanoparticles were effective on all *Candida* species and after photodynamic therapy, the MICs of the yeasts decreased. Antifungal effect of silver nanoparticles on *C. albicans* was reported in the literature [ 38 , 39 ]; however, there are limited data about its effect on non-albicans *Candida* species. In the present study, the antifungal effect of the combination on non-albicans *Candida* species (*C. glabrata* and *C. parapsilosis*) increased after photodynamic therapy which is important in the clinic since these species are more resistant to antifungal agents, compared to *C. albicans* [ 1 , 2 ]. 

In biofilm formation, microorganisms can attach to a surface and grow on by secreting extracellular polysaccharides. Biofilms are more resistant to antibiotics than planktonic cells and have a significant role in public health. Bacteria are the major micro-organisms that make biofilms; however, the fungi are becoming an increasingly common organism, especially *C. albicans* [ 14 ]. Evaluation of susceptibility of biofilms to antimicrobial agents cannot be determined by standard microdilution testing which is used for the planktonic cells since photons or anti-bacterial agents affect the surface of the biofilms and cannot penetrate the depth of the biofilm. 

In the present study, the viability of *C. albicans* biofilm declined about 15%after irradiation with methylene blue as a photosensitizer; however, it was not effective on *C. glabrata* and *C. parapsilosis* with or without irradiation. In the study carried out by Sousa et al., no reduction in CFU/mL of *C. albicans* was reported after the use of methylene blue in the absence of irradiation, which was consistent with the results of this study [ 32 ]. 

Silver nanoparticles were effective on the viability of all *Candida* species in this study and the viability of the yeasts decreased after photodynamic therapy. Addition of silver nanoparticles to methylene blue in photodynamic therapy induced more potent antifungal activity on the biofilm of these species. Dovigo et al. observed a significant decrease in the viability of *C. albicans* and *C. glabrata* biofilm after photodynamic therapy [ 37 ]. Mima et al. reported that photodynamic therapy resulted in the reduction of CFU/mL of *C. albicans*, *C. glabrata*, C. tropicalis, C. dubliniensis, and C. krusei [ 40 ]. Moreover, inhibition of *C. albicans* biofilm formation by silver nanoparticles in the surgical sites was reported in the literature [ 41 ]. 

There are some limitations for in vivo application of photosensitizers in teeth, lips, buccal mucosa, tongue, and removable or fixed prosthetics due to staining; therefore, non-dye photosensitizers may be more appropriate for intraoral use of photodynamic therapy. 

## Conclusion

Based on the results, photodynamic therapy with the photosensitizers was effective in reducing the number of *Candida* species. In addition, the fungicidal effect of photodynamic therapy was strain- and photosensitizer-dependent. Methylene blue, silver nanoparticle, and their combination were effective photosensitizers with antifungal property against *Candida* species. 

## Authors’ contribution

In this research, F. L, P. B, M. Y and P. H. designed the study, acquiesced, analyzed, critical revision of it for important intellectual content and interoperated the data. All the authors participated in the preparation of the draft of the manuscript and approved its final version.

## Financial disclosure

No financial interests related to the material of this manuscript have been declared.
